# Chromatin remodeling and epigenetic regulation in chronic kidney disease

**DOI:** 10.3389/fgene.2026.1781322

**Published:** 2026-01-14

**Authors:** Jinzi Liang, Chao Wan

**Affiliations:** Cancer Center, Union Hospital, Tongji Medical College, Huazhong University of Science and Technology, Wuhan, China

**Keywords:** chromatin remodeling, chronic kidney disease, DNA methylation, epigenetic, histone modification

## Abstract

The progression of chronic kidney disease (CKD) stems from persistent maladaptive alterations in renal cellular gene expression, processes governed by dynamic epigenetic regulation. The dynamic regulation of chromatin encompasses structural changes, condensation, altered accessibility, and ATP-dependent remodeling, playing a pivotal role in CKD progression. Dysregulation of these processes is increasingly implicated in key pathogenic events such as fibroblast activation and inflammatory responses. Recent advances in multi-omics approaches have provided crucial insights into the composition, mechanisms, and dysregulation of chromatin states within diseased kidneys. In this review, we summarize the current understanding of chromatin dynamics and remodeling in CKD, emphasizing their role in disease progression and their interplay with other epigenetic layers. We further discuss how these mechanisms contribute to establishing pathogenic transcriptional memory and highlight emerging therapeutic opportunities.

## 1 Introduction

Chronic kidney disease (CKD) is emerging as a rapidly escalating global health crisis and a major contributor to mortality worldwide (Tampe and Zeisberg, 2014; Rockey et al., 2015; Romagnani et al., 2017). Existing treatment strategies prove largely ineffective, primarily serving to delay disease progression rather than halt or reverse its course (Tampe and Zeisberg, 2014). The prevalence of CKD increases sharply with age and constitutes a major independent risk factor for cardiovascular disease (Bansal et al., 2019; Zhang et al., 2024). Diabetes mellitus, hypertension, glomerulonephritis, and ischemic lesions can all induce persistent, self-perpetuating pathological alterations in the nephron (Oda and Yoshizawa, 2021; Burnier and Damianaki, 2023; Rivetti et al., 2023; Beamish et al., 2024). These manifest as progressive loss of nephrons, capillary rarefaction, and persistent inflammation, ultimately leading to extensive tissue fibrosis. Renal fibrosis frequently accompanies an established autonomous, maladaptive cellular program (Rockey et al., 2015). Even when the underlying cause is treated, the disease often continues to progress, serving as a histological marker of irreversible functional decline.

The persistence of inflammation and fibrosis indicates a fundamental and stable reprogramming of gene expression within renal cells (Nangaku et al., 2017; Miguel et al., 2025; Mimura et al., 2025). This signifies a shift in cellular state from transient activation to a long-term pathogenic phenotype. Such enduring phenotypic conversion cannot be fully explained by genetic predisposition or the ongoing presence of primary injury; it suggests the existence of regulatory mechanisms establishing a form of “cellular memory” that fixes pathological gene expression patterns (Kato and Natarajan, 2019). Epigenetics, by regulating gene expression dynamics and heritability without altering DNA sequences themselves, provides a crucial complementary layer to gene expression control (Cavalli and Heard, 2019; Webster and Phillips, 2025). Epigenetic regulation encompasses chromatin remodeling, histone modifications, DNA methylation, and RNA modifications (Jones, 2012; Barbieri and Kouzarides, 2020; Centore et al., 2020; Yao et al., 2024). As a key regulatory mechanism for cellular responses to metabolic stress, inflammatory cytokines, and oxidative stress damage, epigenetics translates these stimuli into enduring transcriptional outputs (Gibson et al., 2022; García-Giménez et al., 2025; Miguel et al., 2025). This leads to abnormal fate transitions in renal tubular epithelial cells, fibroblasts, and immune cells, subsequently forming pathological states characterized by chronic inflammation and excessive extracellular matrix production (Huang B. et al., 2020; Li H. et al., 2024). Unlike static genetic mutations, epigenetic marks are inherently reversible, offering a unique therapeutic opportunity to reset pathogenic gene expression and break the self-perpetuating cycle of chronic kidney disease progression.

Within the broader epigenetic regulatory system, chromatin dynamics have emerged as a central and upstream mechanism that physically governs the transcriptional potential of the genome (Gibson et al., 2019; Ahmad et al., 2022). This concept extends beyond ATP-dependent chromatin remodeling to encompass the overall structural organization, accessibility, and functional state of chromatin (Centore et al., 2020; Baxter et al., 2023). Chromatin remodelers are large, multi-subunit complexes that utilize ATP hydrolysis to slide, evict, or restructure nucleosomes, thereby fundamentally determining which genomic regions become accessible to the transcriptional machinery. These programs orchestrate fibroblast activation, sustain inflammatory responses, and arrest reparative processes across diverse renal cell types (Muto et al., 2021; Gisch et al., 2024). Targeting this dynamic chromatin regulatory hub holds promise for developing therapies capable of modifying disease progression by resetting maladaptive cellular memory in CKD.

This review systematically outlines chromatin-centric epigenetic regulatory mechanisms in chronic kidney disease (CKD). It first outlines key elements of chromatin dynamics regulation, including the dynamic organization of chromatin and its dysregulation mechanisms in disease. Subsequently, it focuses on core epigenetic interactions, detailing how histone modifications and broader chromatin remodeling establish pathogenic transcriptional memory through bidirectional cross-regulation. This chromatin-centred perspective is further integrated into a broader epigenetic framework. By elucidating these interconnected mechanisms, we highlight the therapeutic potential of targeting chromatin regulation to halt disease progression, offering novel insights for developing innovative treatment strategies for chronic kidney disease.

## 2 Chromatin dynamics in CKD: an overview

### 2.1 Chromatin dynamics: components and regulatory layers

**FIGURE 1:**
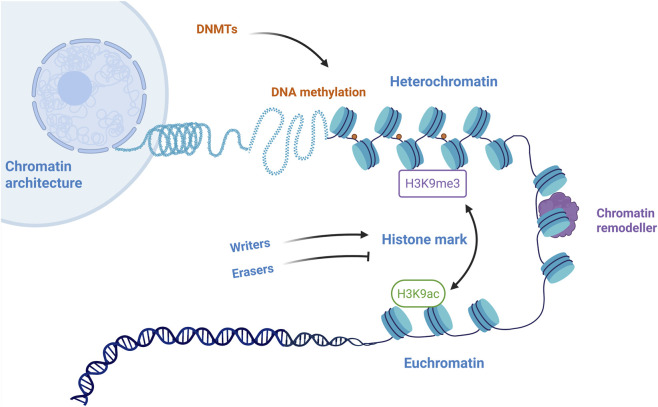
Coordinated regulation of chromatin architecture by DNA methylation and histone modifications. This schematic illustrates the synergistic interplay between key epigenetic layers that determine chromatin states. DNA methylation, catalyzed by DNA methyltransferases (DNMTs), is associated with the formation of transcriptionally silent heterochromatin. This repressive environment is further characterized and maintained by the presence of the H3K9me3 histone mark. Conversely, on the right, the active histone mark H3K9ac is a hallmark of open, transcriptionally permissive euchromatin. The dynamic transition between these states is physically executed by chromatin remodelers, which alter nucleosome positioning and accessibility. Writers and Erasers represent the enzymes that add or remove chemical groups (e.g., methyl, acetyl) on DNA or histones, respectively, thereby directing the chromatin conformation. This integrated model highlights how the crosstalk between DNA methylation, specific histone marks, and chromatin remodeling collectively governs gene expression patterns, a central mechanism dysregulated in chronic kidney disease progression.

Chromatin dynamics refer to the collective, reversible processes that govern the structure, composition, and functional state of chromatin, ultimately determining cellular identity and responses to stimuli (Chen et al., 2024). The foundational layer is chromatin structure and accessibility (Figure 1). Over 2 m of DNA are packaged within the nucleus by wrapping around histone octamers to form nucleosomes, which further fold into higher-order architectures. The functional state of this structure exists on a spectrum between transcriptionally permissive euchromatin and transcriptionally silent heterochromatin. A key mechanism regulating this access is ATP-dependent chromatin remodeling, carried out by complexes like SWI/SNF, ISWI, CHD, and INO80, which physically slide, evict, or restructure nucleosomes (Eustermann et al., 2024). In endometrial carcinoma, the loss of ARID1A and ARID1B subunits disrupts the assembly of the classical BAF (cBAF), thereby altering pathogenic chromatin landscapes and transcriptional programs to promote dedifferentiation and tumor progression (St Laurent et al., 2025). There is evidence that in response to viral infection, the chromatin remodeler SMARCA4 and the transcriptional regulator BRD4 are dynamically induced and repositioned across the genome in airway basal epithelial cells. This coordinated redistribution enables the coupling of intrinsic antiviral defense programs with cellular plasticity pathways, ultimately promoting cell survival, epithelial repair, and mucosal regeneration following injury (Xu and Brasier, 2025). Another crucial layer is the chemical modification of histones, where post-translational marks on histone tails or globular domains act as signals that recruit effector proteins to either open or compact chromatin. Tian et al. demonstrate that in the cabbage beetle, the chromatin remodeler PBAP cooperates with the SET1/COMPASS complex to regulate summer diapause entry (Tian et al., 2025). Wang et al. show that antiandrogen therapy in prostate cancer reconfigures the chromatin regulatory network by enabling the histone reader ZMYND8, which recognizes the H3K4me1–H3K14ac modification, to interact with the chromatin remodeler SWI/SNF, thereby activating a neuroendocrine differentiation program (Wang et al., 2025). Furthermore, the incorporation of histone variants in place of canonical histones adds another dimension of regulation, influencing DNA repair, transcriptional activation, or repression (Martire and Banaszynski, 2020). Jamge et al. shows that specific H2A variants are strongly associated with distinct combinations of histone modifications, effectively partitioning the genome into chromatin subdomains. Furthermore, they demonstrate that the chromatin remodeler DDM1 is required for the proper exchange of H2A variants, such as the deposition of H2A.W over H2A.Z in constitutive heterochromatin, which in turn regulates the organization of these chromatin states (Jamge et al., 2023). Finally, DNA methylation at CpG islands, particularly on gene promoters, typically reinforces a closed chromatin state and stable gene silencing. In a study on the purple sea urchin *Strongylocentrotus purpuratus*, researchers investigated how environmentally induced changes in DNA methylation affect gene regulation. They found that the impact of differential gene body methylation on expression was significantly stronger for genes with low chromatin accessibility at their transcriptional start sites, and that the direction of this effect depended on the gene’s baseline expression level (Bogan et al., 2023). In summary, chromatin structure, remodeling, histone modifications, variants, and DNA methylation form a highly integrated and cooperative system. Their continuous crosstalk is not merely incidental but is essential for achieving the precise and adaptable gene regulation that governs cell fate and environmental response.

### 2.2 Dysregulation of chromatin dynamics in CKD

In chronic kidney disease, the coordinated system of chromatin dynamics becomes fundamentally disrupted, driving the transition from adaptive healing to maladaptive, self-perpetuating fibrosis. This dysregulation manifests as global and cell-type-specific alterations across multiple regulatory layers. On a global scale, fibrotic kidneys exhibit genome-wide alterations in chromatin conformation, indicating a broad breakdown in higher-order nuclear architecture. Supporting this, integrated epigenomic analysis of human kidneys reveals that the transition to a pro-fibrotic state in tubular cells is driven by a specific rewiring of chromatin accessibility, orchestrated by transcription factors such as ELF3, KLF6, and KLF10, which activate injury-response gene programs (Gisch et al., 2024). Research also shows that after kidney injury, the pattern of chromatin accessibility in tubular cells changes early. These specific changes determine whether the cells later activate gene programs for healthy repair or for fibrosis. The transcription factor RXRα is important for maintaining the healthier, protective pattern of chromatin accessibility (Gisch et al., 2024). Integrating snATAC-seq with Hi-C analysis in human diabetic kidney disease has further delineated this pathological remodeling. These studies identified a pro-fibrotic and inflammatory injured proximal tubule cell state, characterized by a distinct chromatin accessibility landscape. Crucially, the transcription factor BACH1 was pinpointed as a core regulator of this state. Multi-omics integration revealed that BACH1 binding regions exhibited increased chromatin contact frequency with the promoters of its target genes in diseased kidneys, directly linking alterations in long-range 3D chromatin architecture to the activation of a specific pathogenic gene program driving tubular injury and fibrosis (Abedini et al., 2024; Eun et al., 2024). Parallel to these large-scale architectural changes, the molecular composition and modification landscape of chromatin are also profoundly altered in CKD. In renal ischemia-reperfusion injury, early evidence indicated the recruitment of histone-modifying enzyme SET1 and chromatin remodeler BRG1 to pro-fibrotic gene promoters, establishing active histone marks like H3K4me3 (Zager and Johnson, 2009). In renal fibroblasts, BRG1 is essential for their activation into matrix-producing myofibroblasts in response to angiotensin II or high glucose, and its genetic or pharmacological inhibition attenuates fibrosis in mouse models of chronic kidney disease and diabetic nephropathy (Hong et al., 2024; Wu et al., 2024). In tubular epithelial cells, BRG1 promotes a pro-fibrotic senescent state by activating the Wnt/β-catenin pathway and suppressing protective autophagy (Gong et al., 2021). Furthermore, in diabetic kidney disease, the long non-coding RNA lncMGC orchestrates a pro-fibrotic response by physically interacting with chromatin remodelers like SMARCA5. This lncRNA-remodeler complex enhances chromatin accessibility at specific loci, thereby amplifying the expression of transforming growth factor-β (TGF-β)–induced fibrotic genes in renal cells (Kato et al., 2023). Thus, the disruption of chromatin dynamics is a central mechanism that establishes and maintains the maladaptive cellular phenotypes underlying CKD progression.

## 3 Crosstalk between histone modifications and chromatin remodeling in CKD

The sustained changes in gene expression that drive CKD progression result from the coordinated interaction of different epigenetic regulatory layers. A key interaction is the bidirectional relationship between histone modifications and changes in chromatin structure and accessibility. This interplay helps establish and maintain the pathogenic gene expression programs that underlie fibrosis, inflammation, and failed repair in the diseased kidney.

### 3.1 Histone modifications in CKD: drivers of cellular reprogramming

In CKD, persistent injurious stimuli rewire the renal epigenome through distinct histone modification patterns, which act as central drivers of cellular reprogramming across diverse cell types. Dysregulated histone methylation plays a pivotal role. The balance between activating and repressive methyl-marks is frequently disrupted. For instance, the upregulation of pro-fibrotic genes in diabetic nephropathy is associated with a loss of the repressive mark H3K27me3 and a concurrent gain of the active mark H3K4me2 at their loci, a change linked to increased expression of the demethylase KDM6A (Komers et al., 2013). Conversely, the methyltransferase EZH2 catalyzes H3K27me3 to silence protective genes. By repressing PTEN via this mark, EZH2 activates pro-fibrotic signaling that drives epithelial-mesenchymal transition and fibrosis during acute kidney injury (AKI)-to-CKD progression (Zhou et al., 2023). On the other hand, enzymes like MLL1 promote tubular apoptosis by catalyzing the activating mark H3K4me3 (Zhang et al., 2022). Furthermore, the transcription factor DACH1 protects podocytes in diabetic kidney disease by recruiting the PTIP complex to specific gene promoters. This recruitment reduces local H3K4me3 levels and represses transcription, whereas loss of DACH1 leads to epigenetic derepression and increased injury susceptibility (Cao et al., 2021). A significant shift in histone acetylation balance is also evident, often characterized by increased deacetylase activity that suppresses repair pathways, while targeted hyperacetylation by enzymes like PCAF at pro-inflammatory gene loci sustains a chronic inflammatory milieu (Huang et al., 2015). There is evidence that the transcription factor ATF3, which is upregulated in CKD, recruits histone acetyltransferases to maintain H3K27ac levels at target genes, thereby driving a pro-fibrotic transcriptional program (Yang et al., 2025). Additionally, in cardiorenal syndrome, the uremic toxin high phosphate increases H3K9 acetylation to upregulate the transcription factor IRF1, which in turn suppresses PGC1α and disrupts myocardial energy metabolism, linking renal dysfunction to cardiac pathology (Huang Y. et al., 2020). Furthermore, the metabolic disturbances inherent to CKD, such as a glycolytic shift, fuel emerging modifications. Histone lactylation, driven by PFKFB3 and lactate accumulation, and histone crotonylation, dependent on ACSS2, are implicated in activating NF-κB-driven inflammation and promoting cellular senescence, respectively (Li L. et al., 2024; Wang et al., 2024). These modifications collectively establish a pathogenic epigenetic landscape that directs persistent transcriptional changes in a cell-type-specific manner, promoting tubular senescence and partial epithelial-mesenchymal transition (EMT), sustaining fibroblast activation, and stabilizing a pro-inflammatory immune phenotype. This reprogramming ultimately solidifies the irreversible progression of CKD.

### 3.2 Interplay between histone marks and chromatin remodeling in CKD

The deposition of specific histone marks serves as a primary mechanism for dynamically regulating higher-order chromatin structure, directly influencing its condensation state and functional accessibility, which in turn dictates transcriptional outcomes in CKD. This relationship is fundamental to establishing pathogenic epigenetic memory. Repressive modifications such as H3K27me3 and H3K9me3 are intrinsically linked to the formation of condensed, transcriptionally silent heterochromatin. Yu et al. show that the dynamic removal of this repressive mark is equally critical. Following acute kidney injury, activation of the histone demethylase JMJD3, which catalyzes H3K27me3 removal, promotes an open chromatin state that facilitates the expression of Klotho and BMP-7, thereby supporting tubular cell survival and regeneration (Yu C. et al., 2024). Additionally, a pathogenic feed-forward loop involving the repressive mark H3K27me3 has been identified in cisplatin-induced AKI. Here, upregulation of the transcription factor AhR enhances expression of the methyltransferase EZH2, which in turn deposits H3K27me3 to epigenetically reinforce AhR expression. This reciprocal AhR-EZH2 regulation drives tubular epithelial cell senescence, demonstrating how a repressive histone mark can be locked into a self-amplifying circuit to perpetuate injury. Conversely, activating marks function to decompact chromatin. Histone acetylation, by neutralizing the positive charge on lysines, weakens histone-DNA interactions and promotes an open, accessible euchromatin state. For example, Lu et al. show that in diabetic kidney disease, loss of the deacetylase SIRT7 results in sustained H3K18 hyperacetylation at specific loci. This altered histone landscape facilitates an open chromatin state at the SDC1 promoter, where SIRT7 normally cooperates with HIC1 to maintain repression (Lu et al., 2024). For instance, H3S10ph during endothelial activation is associated with localized chromatin relaxation at the VCAM1 locus, facilitating inflammatory responses (Alghamdi et al., 2018). Similarly, the deacetylase SIRT6 exerts a protective role in podocytes. Sirt6 deficiency leads to increased histone H3K9 acetylation, which promotes an open chromatin configuration at the promoters of Notch1 and Notch4 genes. This epigenetic derepression activates Notch signaling, contributing to podocyte injury and proteinuria in diabetic kidney disease (Liu et al., 2017). This reciprocal regulation between the chemical histone code and the physical chromatin state creates a stable, self-reinforcing cycle that is challenging to reverse. Breaking this cycle presents novel therapeutic opportunities, such as developing small molecules that target the reader domains of chromatin-associated proteins to disrupt the interpretation of the histone code, or employing dual-target strategies against key writers and the chromatin remodeling apparatus to reset the pathological epigenetic landscape. Notably, this chromatin-centric regulatory hub does not function in isolation; its output is profoundly integrated with and reinforced by other fundamental epigenetic mechanisms, including DNA methylation and RNA modifications, which collectively establish the robust pathological memory characteristic of progressive CKD.

## 4 Other epigenetic regulations in chronic kidney diseases

In addition to chromatin remodeling and alterations in nuclear architecture, other fundamental layers of epigenetic regulation, including DNA methylation, non-coding RNAs, and RNA chemical modifications, play indispensable roles in the pathogenesis and progression of CKD. These mechanisms collectively mediate sustained transcriptional and post-transcriptional reprogramming in renal cells in response to injury and the pathological microenvironment.

### 4.1 DNA methylation in CKD

DNA methylation, involving the covalent addition of a methyl group to cytosine residues in CpG dinucleotides, is a key epigenetic mark associated with long-term transcriptional silencing (Jones et al., 2015). Extensive research has established that dynamic alterations in DNA methylation patterns are fundamental to establishing and maintaining a pro-fibrotic phenotype in renal cells during CKD (Rysz et al., 2022).

CKD progression is characterized by both global DNA hypomethylation and promoter-specific hypermethylation (Smyth et al., 2014; Yang et al., 2016). Global hypomethylation may contribute to genomic instability and aberrant expression of normally silenced genes. In contrast, targeted hypermethylation frequently occurs at the promoters of critical protective genes. A canonical example is the hypermethylation and silencing of the RASAL1 gene, which encodes a negative regulator of the Ras GTPase pathway. This epigenetic inactivation leads to sustained Ras signaling, driving fibroblast activation and excessive extracellular matrix deposition—a central event in renal fibrosis (Bechtel et al., 2010; Tampe et al., 2014; Tampe et al., 2017). This repression extends to other vital protective pathways. The promoter hypermethylation of KLOTHO, a gene with anti-aging and renal protective functions, is a well-documented event in CKD; its silencing disrupts phosphate metabolism (Sun et al., 2012; Yin et al., 2017). Similarly, epigenetic downregulation of genes within the renin-angiotensin system (RAS) and those involved in cellular metabolism further accelerates disease progression.

DNA methylation serves as a molecular interface linking the uremic metabolic milieu to persistent gene expression changes. Metabolic disturbances in CKD, such as elevated homocysteine, can impair DNA methyltransferase (DNMT) activity and shift global methylation patterns (Yang et al., 2016). Furthermore, environmental stimuli like hyperglycemia can induce specific, heritable methylation changes that lock renal cells into a profibrotic state. The pharmacological reversibility of DNA methylation marks presents a promising therapeutic avenue (Ito et al., 2010). Preclinical studies demonstrate that DNMT inhibitors 5-aza-2′-deoxycytidine can reverse promoter hypermethylation, reactivate silenced anti-fibrotic genes, and significantly attenuate renal fibrosis in animal models (Chang et al., 2016). This positions DNA methylation not only as a pathogenic driver but also as a viable target for epigenetic therapy in CKD.

### 4.2 Non-coding RNAs in CKD

Alongside DNA methylation, non-coding RNAs represent a diverse and crucial class of regulatory molecules whose dysregulation is a hallmark of various CKD etiologies. Among these, microRNAs (miRNAs) have been extensively studied for their role in post-transcriptional regulation. Certain miRNAs, such as the upregulated miR-21, miR-192, and miR-433, function as potent drivers of fibrosis by directly repressing anti-fibrotic mRNAs like SMAD7 and ZEB1/2, thereby promoting extracellular matrix accumulation (Chung et al., 2010; Zhong et al., 2011; Lan, 2012; Xiong et al., 2012). Conversely, the downregulation of protective miRNA families, including miR-29 and miR-200, releases their repression on collagen genes and EMT inducers, further exacerbating tissue scarring (Tang et al., 2013; Lv et al., 2018). Due to their notable stability in blood and urine, specific circulating miRNAs are also being actively investigated as promising non-invasive biomarkers for CKD diagnosis and tracking disease progression.

Beyond miRNAs, long non-coding RNAs (lncRNAs) contribute to CKD pathogenesis through diverse mechanisms. For instance, upregulated lncRNAs like MALAT1 and Arid2-IR promote renal fibrosis by scaffolding chromatin modifiers or sequestering anti-fibrotic miRNAs (Zhou et al., 2015; Li et al., 2019), whereas the downregulation of protective lncRNAs such as MEG3 disrupts normal cellular signaling (Yiu et al., 2023). The regulatory network is further complicated by emerging roles for other ncRNA species, including circular RNAs (circRNAs) and piwi-interacting RNAs (piRNAs), which primarily function through mechanisms like miRNA sponging and transcriptional control.

### 4.3 RNA modification in CKD

Beyond DNA methylation and non-coding RNAs, the dynamic and reversible N^6^-methyladenosine (m^6^A) RNA modification constitutes a crucial layer of post-transcriptional epigenetic regulation in CKD (Luan et al., 2023). As the most prevalent internal modification in eukaryotic mRNA, m^6^A deposition, removal, and recognition—catalyzed by writers (e.g., METTL3/METTL14), erasers (e.g., FTO, ALKBH5), and readers (e.g., YTHDF family, IGF2BPs)—profoundly influence RNA splicing, stability, localization, and translation (Jiang et al., 2021). Dysregulation of this epigenetic machinery is a central driver of maladaptive gene expression, promoting renal fibrosis, inflammation, and cellular injury across the CKD spectrum.

In renal fibrosis models, such as unilateral ureteral obstruction, the writer METTL3 is frequently upregulated (Meng et al., 2020). It facilitates fibrosis by promoting the maturation of pri-miR-21-5p and stabilizing the lncRNA MALAT1 (Liu et al., 2020; Liu et al., 2021), thereby activating the SPRY1/ERK/NF-κB inflammatory pathway and the miR-145/FAK pro-fibrotic axis, respectively. Conversely, downregulation of the eraser ALKBH5 exacerbates epithelial-mesenchymal transition (Ning et al., 2020). In diabetic kidney disease, m^6^A exerts cell-type-specific effects: in podocytes, METTL3 can modulate NLRP3 inflammasome-mediated pyroptosis or stabilize TIMP2 mRNA to promote injury (Jiang et al., 2022); in glomerular endothelial cells, METTL14 suppresses the protective factor α-Klotho via m^6^A modification (Li et al., 2021). Furthermore, in autosomal dominant polycystic kidney disease, a hyperactive METTL3-m^6^A axis enhances the translation of pro-proliferative mRNAs like c-Myc, accelerating cyst growth (Ramalingam et al., 2021). Importantly, distinct m^6^A regulator signatures in lupus nephritis and altered m^6^A levels in immune cells from CKD patients highlight its role in immune dysfunction and diagnostic potential.

The reversible nature of m^6^A and its specific dysregulation position it as a promising therapeutic target. Preclinical interventions targeting METTL3 or ALKBH5 have shown efficacy in attenuating renal fibrosis, inflammation, and cyst expansion. Thus, m^6^A RNA methylation represents an integral and targetable component of the epigenetic network driving CKD pathogenesis.

Beyond m^6^A, other RNA modifications constitute an additional layer of epitranscriptomic regulation with direct relevance to kidney pathology. Among these, 5-methylcytosine (m^5^C) has been mechanistically implicated across the spectrum of kidney injury. In acute settings, the m^5^C ‘writer’ NSUN3 stabilizes pro-inflammatory TIFA mRNA, exacerbating tubular injury in sepsis-associated AKI (Zhang et al., 2025). In chronic immune-mediated disease, TRDMT1-driven m^5^C in B cells promotes pathogenic IgA class-switch recombination, a key process in IgA nephropathy (Yu S. et al., 2024). Further expanding this landscape in metabolic disease, the m^5^C ‘eraser’ TET2 is deficient in diabetic nephropathy. TET2 loss leads to hyper-methylation and dysregulation of Bcas3 mRNA, impairing mitophagy and exacerbating podocyte injury, whereas TET2 restoration is protective (Ma et al., 2025). These studies collectively demonstrate that m^5^C is a dynamic and cell type-specific regulator, influencing inflammation, immune response, and cellular metabolism in kidney disease. While the roles of ac^4^C, Ψ, and other modifications remain largely unexplored in CKD, the established regulatory logic of the epitranscriptome suggests they hold broad, untapped potential. A comprehensive mapping of these modifications will be crucial for a complete understanding of post-transcriptional dysregulation in CKD progression.

## 5 Conclusion

Chronic kidney disease progression is underpinned by a stable maladaptive reprogramming of gene expression in renal cells—a form of pathogenic “cellular memory.” As this review has detailed, this memory is largely encoded and maintained within the dynamic landscape of chromatin. The dysregulation of chromatin dynamics, encompassing alterations in higher-order architecture, nucleosome positioning, accessibility, and the associated histone modification landscape, acts as a central regulatory hub that integrates injurious signals to establish and perpetuate fibrotic and inflammatory transcriptional programs.

The interplay between histone modifications and chromatin remodelers forms a self-reinforcing cycle. Repressive marks like H3K27me3 compact chromatin to silence protective genes, while activating marks such as H3K27ac and H3K9ac promote an open configuration to drive pro-fibrotic transcription. Chromatin remodelers like BRG1 and lncRNA-associated complexes physically execute these structural changes, locking in the pathological state. This chromatin-centric dysregulation is further integrated with other epigenetic layers, including DNA hypermethylation of genes like RASAL1, dysregulation of non-coding RNAs, and altered RNA m^6^A modifications, creating a robust and multifaceted epigenetic barrier to recovery.

Critically, the very reversibility of these epigenetic marks presents a unique therapeutic opportunity. Unlike genetic mutations, the pathological chromatin state can, in principle, be reset. Emerging strategies aim to disrupt this cycle by targeting its key components: small-molecule inhibitors of chromatin remodelers, histone-modifying enzymes, or the readers of epigenetic marks. Furthermore, modulating upstream signals that drive epigenetic dysregulation or employing combination therapies to target multiple epigenetic layers simultaneously may enhance efficacy. As our understanding of the renal epigenome deepens, particularly through single-cell multi-omics, the precision of these interventions will improve.

In conclusion, viewing CKD through the lens of chromatin dynamics provides a unifying framework for its persistent and progressive nature. Targeting the reversible epigenetic machinery that governs chromatin states offers a promising frontier for developing disease-modifying therapies that move beyond mere symptom management towards resetting the pathogenic cellular memory and halting disease progression. However, to translate this promise into viable therapies, key challenges must be overcome, including achieving cell-type specificity in targeting, ensuring the long-term safety of modulating global epigenetic machinery, and defining the optimal therapeutic window for intervention. Furthermore, fundamental questions remain: how are the different layers of epigenetic regulation dynamically coordinated *in vivo*, and can distinct ‘epigenetic signatures’ predict individual trajectories of CKD progression? Addressing these challenges and questions will be essential to advance epigenetic therapeutics from concept to clinical reality.
